# A novel biclustering algorithm of binary microarray data: BiBinCons and BiBinAlter

**DOI:** 10.1186/s13040-015-0070-4

**Published:** 2015-11-30

**Authors:** Haifa Ben Saber, Mourad Elloumi

**Affiliations:** 1 Latice laboratory, ENSIT, Tunis Time université, Tunis, Tunisia; 2 Latice laboratory, Ensit, Tunis Université tunis el manar, Tunis, Tunisia

**Keywords:** Biclustering, Algorithm, Evaluation function, Microarray data analysis

## Abstract

The biclustering of microarray data has been the subject of a large research. No one of the existing biclustering algorithms is perfect. The construction of biologically significant groups of biclusters for large microarray data is still a problem that requires a continuous work. Biological validation of biclusters of microarray data is one of the most important open issues. So far, there are no general guidelines in the literature on how to validate biologically extracted biclusters. In this paper, we develop two biclustering algorithms of binary microarray data, adopting the *Iterative Row and Column Clustering Combination* (IRCCC) approach, called *BiBinCons* and *BiBinAlter*. However, the *BiBinAlter* algorithm is an improvement of *BiBinCons*. On the other hand, *BiBinAlter* differs from *BiBinCons* by the use of the *EvalStab* and *IndHomog* evaluation functions in addition to the *CroBin* one (Bioinformatics 20:1993–2003, 2004). *BiBinAlter* can extracts biclusters of good quality with better *p-values*.

## Introduction

DNA microarray technology is a revolutionary tool enabling the measurement of expression levels of thousands of genes in a single experiment under diverse experimental conditions. This technology allows us to obtain big raw data that can provide a wealth of information on the concerned genes. It proved to be a valuable tool for many biological and medical applications. Indeed, microarray data analysis is a crucial step for these applications in order to extract pertinent biological knowledge embedded in these large masses of data. However, the extraction process of this knowledge is far from being trivial. From here comes the necessity to adopt *data mining* techniques. Many of these techniques were applied to these data in order to extract pertinent biological knowledge. Among the techniques that are used, we mention those of *clustering* [1]. Indeed, by making a *clustering*, we consider that all the genes of a group can have a similar behavior under all the conditions. However, there are genes that have a similar behavior only under a subset of conditions. Hence, clustering is too simplistic to detect such cases [1]. Another more interesting technique, called *biclustering* [2], allows to identify groups of genes that have a similar behavior only under a subset of conditions.

In this paper, we develop new biclustering algorithms of microarray data. These data are usually coded by a data matrix *M*(*I,J*), where the *i*^*th*^ row, *i*∈*I*={1,2,…,*n*}, represents the *i*^*th*^ gene, the *j*^*th*^ column, *j*∈*J*={1,2,…,*m*}, represents the *j*^*th*^ condition and the cell *M*[*i,j*] represents the expression level of the *i*^*th*^ gene under the *j*^*th*^ condition.

The main objective is then to identify groups of genes that are coherent under groups of conditions, these groups are called *biclusters*. Genes belonging to the same bicluster have close biological functions. Let’s note that, in its general form, the biclustering problem is NP-hard [2].

The rest of this chapter is organized as follow: In the second section, we introduce some preliminaries. In the third section, we present the *BiBinCons* algorithm. In the fourth section, we present the *BiBinAlter* algorithm. In the fifth section, we present an illustrative example and an experimental study. Finally, we present the conclusion of this paper.

## Preliminaries

As we said in the introduction, the biclustering algorithms that we present in this paper are based on *CroBin* [1] function for the evaluation of a group of biclusters. So, let’s present some preliminaries related to this function. Let *I*={1,2,…,*n*} be a set of indices of *n* genes, *J*={1,2,…,*m*} be a set of indices of *m* conditions and $M_{b}(\textit {I,J}) = \left (m_{\textit {ij}}^{b}\right)$, *i*∈*I* and *j*∈*J*, be a binary data matrix associated with *I* and *J*. The biclustering problem of a binary microarray data can be formulated as a minimization of the criterion *W*(*z,w,a*):

(2.1) $$ W(z,w,a)=\sum\limits_{k=1}^{a}\sum\limits_{l=1}^{m}\sum\limits_{i\in z_{k}}\sum\limits_{j\in w_{l}}\left|m_{ij}^{b}-a_{kl}\right|.  $$

where *z*={*z,z*_2_,…,*z*_*g*_} is the matrix defined as a partition of *I* into *g* clusters, i.e. *z*_*i*_ is the cluster number of the *i*^*th*^ row of *M*_*b*_(*I,J*). *w*={*w*_1_,*w*_2_,…,*w*_*h*_} is the matrix defined as a partition of *J* into *h* clusters, i.e. *w*_*i*_ is the cluster number of the *j*^*th*^ column of *M*_*b*_(*I,J*).white.whe

*a* = (*a*_*kl*_) is a *summary matrix* of *M*_*b*_(*I,J*), it is a binary *g*×*h* matrix where *k* (resp. *l*) is the number of clusters on rows (resp. columns) and *a*_*kl*_ is defined by the *m*_*ij*_’s satisfaying the following condition:

(2.2) $$ z_{ik}w_{jl}=1  $$

where *z*_*ik*_=1 if the *i*^*th*^ row of *M*_*b*_(*I,J*) belongs to the *k*^*th*^ cluster of *I* otherwise *z*_*ik*_=0. *w*_*jl*_=1 if the *j*^*th*^ column of *M*_*b*_(*I,J*) belongs to the *l*^*th*^ cluster of *J* otherwise *w*_*jl*_=0.

By using Eq. (2.2), Eq. (2.1) can be reformulated as follows:

(2.3) $$ W(z,w,a)=\sum\limits_{i,j,k,l}z_{ik}w_{jl}\left|m_{ij}^{b}-a_{kl}\right|  $$

By adopting the IRCCC approach, we can make biclustering by minimizing *W*(*z,w,a*) defined by Eq. (2.3) and by fixing either *w* or *z*:

- If *w* is fixed, the minimization is given by:
  (2.4) $$ W(z,a|w)=\sum\limits_{i,k,l}z_{ik}|u_{il}-(|w_{l}|\times a_{kl})|  $$
  where $u_{\textit {il}}=\sum _{j\in w_{l}}m_{\textit {ij}}=\sum _{j}w_{\textit {jl}}m_{\textit {ij}}, \sum \limits _{\textit {i,j,k,l}} z_{\textit {ik}}w_{\textit {jl}}\left |m_{\textit {ij}}^{b}-a_{\textit {kl}}\right |=\underset {i,k}{\sum }z_{\textit {ik}}\underset {j,l}{\sum }w_{\textit {jl}}\left |m_{\textit {ij}}^{b}-a_{\textit {kl}}\right |=\underset {i,k}{\sum }z_{\textit {ik}}\underset {l}{\sum }|u_{\textit {il}}-(|w_{l}|\times a_{\textit {kl}})|$, *u* is a matrix of size |*I*|×*l*.
- If *z* is fixed, the minimization is given by:
  (2.5) $$ W(w,a|z)=\sum\limits_{i,k,l}w_{jl}|v_{jl}-(|z_{k}|\times a_{kl})|\vspace*{-3pt}  $$
  where $v_{\textit {kj}}=\sum _{i\in z_{k}}m_{\textit {ij}}^{b}=\sum _{i}z_{\textit {ik}}m_{\textit {ij}}$, $\underset {\textit {i,j,k,l}}{\sum }z_{\textit {ik}}w_{\textit {jl}}\left |m_{\textit {ij}}^{b}-a_{\textit {kl}}\right |=\sum \limits _{\textit {j,l}} w_{\textit {jl}} \sum \limits _{i,k} z_{\textit {ik}}\left |m_{\textit {ij}}^{b}-a_{\textit {kl}}\right |= \sum \limits _{\textit {i,k}} z_{\textit {ik}} \sum \limits _{l} |v_{\textit {kj}}-(|z_{k}|\times a_{\textit {kl}})|$, *v* is a matrix of size *k*×|*J*|.

### **Remark**.

A colored block in the binary matrix *M*_*b*_(*I,J*) will be represented by a colored cell in the summary matrix *A*, where each colored cell contains the majority binary value in the corresponding colored block, e.g, if the majority of cells in a block in *M*_*b*_(*I,J*) contains 1 then the corresponding cell in *A* contains also 1.

### **Example**.

This example shows a binary data matrix *M*_*b*_(*I,J*) and the corresponding cell in the summary matrix *A*.

**Figure:**
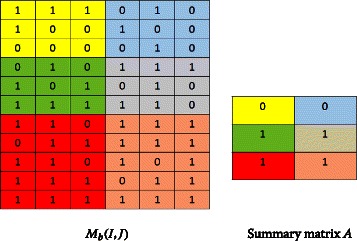

*A*=(0,0;1,1;1,1), i.e., *a*_11_=0,*a*_12_=0;*a*_21_=1, *a*_22_=1;*a*_31_=1,*a*_32_=1.

In the section ‘FIRST IRCCC Algorithm: *BiBinCons*’, we develop two IRCCC algorithms of biclustering of binary microarray data, called respectively *BiBinCons* and *BiBinAlter*.

## FIRST IRCCC Algorithm: *BiBinCons*

Our biclustering algorithm, *BiBinCons* receives as input a binary matrix *M*_*b*_(*I,J*) and gives as output (*z*_*opt*_,*w*_*opt*_,*A*_*opt*_), where *z*_*opt*_ and *w*_*opt*_ are respectively the final clustering of rows and columns of *M*_*b*_(*I,J*), and *A*_*opt*_ is the summary matrix related to *z*_*opt*_ and *w*_*opt*_. To describe more formally our biclustering algorithm, *BiBinCons*, we use the following notations:

*z*_0_ : initial clustering of rows of *M*_*b*_(*I,J*)

*w*_0_ : initial clustering of columns of *M*_*b*_(*I,J*),

*A*_0_ : initial summary matrix related to *z*^0^ and *w*^0^

*z*_*c*_ : current clustering of rows of *M*_*b*_(*I,J*)

*w*_*c*_ : current clustering of columns of *M*_*b*_(*I,J*),

$A_{c}^{'}$ : current intermidate summary matrix related to *z*^*c*^ and *w*^*c*−1^

*A*_*c*_ : current summary matrix related to *z*^*c*^ and *w*^*c*^

*z*_*opt*_ : final clustering of rows of *M*_*b*_(*I,J*)

*w*_*opt*_ : final clustering of columns of *M*_*b*_(*I,J*)

*A*_*opt*_ : final summary matrix related to *z*^*opt*^ and *w*^*opt*^

$A_{c}^{'}$ : intermediate current summary matrix.

**Figure:**
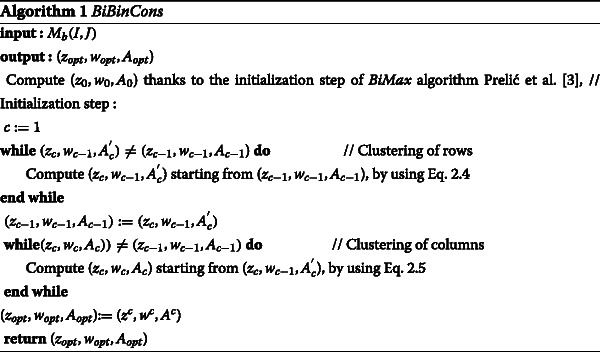

## Second IRCCC Algorithm: *BiBinAlter*

Our biclustering algorithm, *BiBinAlter* receives as input a binary matrix *M*_*b*_(*I,J*) and gives as output (*z*_*opt*_,*w*_*opt*_,*A*_*opt*_), where *z*_*opt*_ and *w*_*opt*_ are respectively the final clustering of rows and columns of *M*_*b*_(*I,J*), and *A*^*opt*^ is the summary matrix related to *z*_*opt*_ and *w*_*opt*_. By adopting *BiBinAlter*, we propose the use of functions defined:

*EvalStab*_*c*_ represents the frequency of 0’s in the current group of biclusters at the *c*^*th*^ iteration. It is defined as follows:

(4.1) $$ EvalStab=\sum\limits_{k,l}\frac{|a_{kl}-(|z_{k}|\times|w_{l}|)|}{|z_{k}||w_{l}|}  $$

*IndHomog*_*c*_ represents the tradeoff between the number of mixed biclusters (containing both 0’s and 1’s) and the total number of biclusters at the *c*^*th*^ iteration. It is defined as follows:

(4.2) $$ IndHomog=\frac{MixedBic}{AllBic}  $$

To describe more formally our biclustering algorithm, *iBinAlter*, we have used the same notations like previous algorithm besides of these notations:

(*EvalStab*_*c*_,*IndHomog*_*c*_): couple to present the frequency of 0’s in the current group of biclusters at the *c*^*th*^ iteration and the tradeoff between the number of mixed biclusters (containing both 0’s and 1’s) and the total number of biclusters at the *c*^*th*^ iteration.

(*EvalStab*_*c*−1_,*IndHomog*_*c*−1_): couple to present the frequency of 0’s in the group of biclusters at the (*c*−1)^*th*^ iteration and the tradeoff between the number of mixed biclusters (containing both 0’s and 1’s) and the total number of biclusters at the (*c*−1)^*th*^ iteration.

$\left (EvalStab_{(c-1)}^{'},IndHomog_{(c-1)}^{'}\right)$: couple to present the frequency of 0’s in the group of biclusters at the intermidate (*c*−1)^′^^*th*^ iteration and the tradeoff between the number of mixed biclusters (containing both 0’s and 1’s) and the total number of biclusters at the intermidate (*c*−1)^′^^*th*^ iteration.

**Figure:**
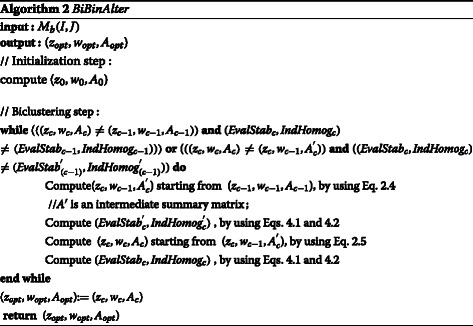

## Illustrative example

Let’s apply the *BiBinAlter* algorithm on the following binary matrix *M*_*b*_(*I,J*):

**Figure:**
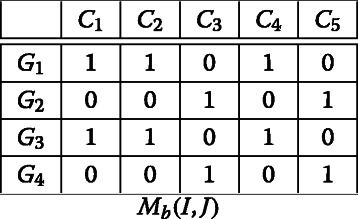

- **Initialization step**

First, we initialize the rows and columns thanks to the initialization step of *BiMax* algorithm Prelić [3] and we compute (*z*_0_,*w*_0_,*A*_0_), we obtain:

*z*_0_=(1,2,2,3), *w*_0_=(1,1,0,0,0), *A*_0_=(1,0;1,1;0,1)

**Figure:**
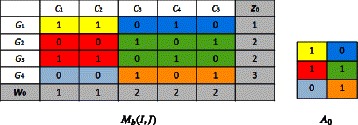

A colored block in the binary matrix *M*_*b*_(*I,J*) will be represented by a colored cell in the summary matrix *A*_0_, where each colored cell contains the majority binary value in the corresponding colored block, e.g, if the majority of cells in a block in *M*_*b*_(*I,J*) contains 1 then the corresponding cell in *A*_0_ contains also 1.

- **Biclustering step:**

**Iteration 1:***c*=1

We compute ($z_{1},w_{0},A_{1}^{'}$) starting from (*z*_0_,*w*_0_,*A*_0_) by using Eq. 2.4, we obtain:

$$\left(z_{1},w_{0},A_{1}^{'}\right)=\left((1,3,2,1),(1,1,2,2,2),(1,1;1,0;0,1)\right) $$

We compute $(EvalStab_{1}^{'},IndHomog_{1}^{'})$ by using Eq. 4.3, we obtain:

$$\left(EvalStab_{1}^{'},IndHomog_{1}^{'}\right)=\left(2,\frac{2}{3}\right) $$

We compute (*z*_1_,*w*_1_,*A*_1_) starting from ($z_{1},w_{0},A_{1}^{'}$), by using Eq. 2.4, we obtain:

$$\left(z_{1},w_{1},A_{1}\right)=\left((1,3,2,1),(2,2,1,2,1),(1,1;1,0;0,1)\right) $$

We compute (*EvalStab*_1_,*IndHomog*_1_), by using Eq. 4.3, we obtain:

$$\left(EvalStab_{1},IndHomog_{1}\right)=\left(1,\frac{2}{6}\right) $$

Since we have

$${\kern30pt} \begin{aligned} ((({z}_{c},{w}_{c},{A}_{c}) & \neq ({z}_{c-1},{w}_{c-1},{A}_{{c}-1}))\,\, \textbf{and}\,\, ({EvalStab}_{c},{IndHomog}_{c})\\ &\left.\left. \neq ({EvalStab}_{c-1},{IndHomog}_{c-1})\right)\right) \end{aligned} $$

**and**

$${\kern30pt} \begin{aligned} (((z_{c},w_{c},A_{c})&\neq(z_{c},w_{c-1},A_{c}^{'})) \,\, \textbf{and}\,\,((EvalStab_{c},IndHomog_{c})\\ &\neq (EvalStab_{(c-1)}^{'},IndHomog_{(c-1)}^{'})) \end{aligned} $$

we make another iteration

**Iteration 2:*c*=2**

We compute ($z_{2},w_{1},A_{2}^{'}$) starting from (*z*_1_,*w*_1_,*A*_1_), by using Eq. 2.4, we obtain:

$$\left(z_{2},w_{1},A_{2}^{'}\right)=\left((2,1,2,3),(2,2,1,2,1),(0,1;1,0,0,1)\right) $$

**Figure:**
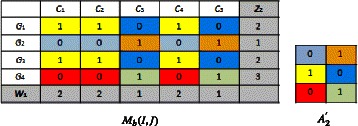

We compute $(EvalStab_{2}^{'},IndHomog_{2}^{'})$, by using Eq. 4.3, we obtain:

$$\left(EvalStab_{2}^{'},IndHomog_{2}^{'}\right)=\left(0,\frac{0}{6}\right) $$

We compute (*z*_2_,*w*_2_,*A*_2_) starting from ($z_{2},w_{1},A_{2}^{'}$), by using Eq. 2.4, we obtain:

$$(z_{2},w_{2},A_{2})=((2,1,2,3),(2,2,1,2,1),(0,1;1,0;0,1)) $$

We compute (*EvalStab*^2^,*IndHomog*^2^), by using Eq. 4.3, we obtain:

$$\left(EvalStab^{2},IndHomog^{2}\right)=\left(0,\frac{0}{6}\right) $$

Since we have

$${\kern30pt} \begin{aligned} (((z_{c},w_{c},A_{c})&\neq(z_{c-1},w_{c-1},A_{c-1}))\,\, \textbf{and}\,\,(EvalStab_{c},IndHomog_{c})\\ &\neq(EvalStab_{c-1}, IndHomog_{c-1}))) \end{aligned} $$

**and**

$${\kern32pt} \begin{aligned} (((z_{c},w_{c},A_{c})&\neq(z_{c},w_{c-1},A_{c}^{'}))\,\,\textbf{and}\,\,((EvalStab_{c},IndHomog_{c})\\ &=(EvalStab_{(c-1)}^{'},IndHomog_{(c-1)}^{'})) \end{aligned} $$

we make another iteration

**Iteration 3:*c*=3**

We compute ($z_{3},w_{2},A_{3}^{'}$) starting from (*z*_2_,*w*_2_,*A*_2_), by using Eq. 2.4, we obtain:

(4.3) $$ \left(z_{3},w_{2},A_{3}^{'}\right)=\left((1,1,1,1),(2,2,1,2,1),(1,1)\right)  $$

**Figure:**
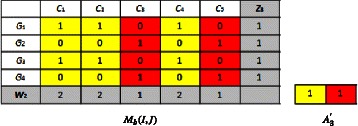

We compute $(EvalStab_{3}^{'},IndHomog_{3}^{'})$, by using Eq. 4.3, we obtain:

$$\left(EvalStab_{3}^{'},IndHomog_{3}^{'}\right)=(1,1) $$

We compute (*z*_3_,*w*_3_,*A*_3_) starting from ($z_{3},w_{2},A_{3}^{'}$), by using Eq. 2.4, we obtain:

$$\left(z_{3},w_{3},A_{3}\right)=((2,1,2,3),(2,2,1,2,1),(0,1;1,0;0,1)) $$

We compute (*EvalStab*^3^,*IndHomog*^3^), by using Eq. 4.3, we obtain:

$$\left(EvalStab^{3},IndHomog^{3}\right)=\left(0,\frac{0}{6}\right) $$

Since we have

$${\kern30pt} \begin{aligned} (((z_{c},w_{c},A_{c})&\neq(z_{c-1},w_{c-1},A_{c-1}))\,\, \textbf{and}\,\, (EvalStab_{c},IndHomog_{c})\\ &\neq (EvalStab_{c-1},IndHomog_{c-1}))) \end{aligned} $$

**and**

$${\kern31pt} \begin{aligned} (((z_{c},w_{c},A_{c})&\neq(z_{c},w_{c-1},A_{c}^{'}))\,\,\textbf{and}\,\, ((EvalStab_{c},IndHomog_{c})\\ &\neq (EvalStab_{(c-1)}^{'},IndHomog_{(c-1)}^{'})) \end{aligned} $$

we make another iteration

**Iteration 4:*c*=4**

We compute ($z_{4},w_{3},A_{4}^{'}$) starting from (*z*_3_,*w*_3_,*A*_3_), by using Eq. 2.4, we obtain:

$$\left(z_{4},w_{3},A_{4}^{'}\right)=((1,2,1,2),(2,2,1,2,1),(1,0;0,1)) $$

**Figure:**
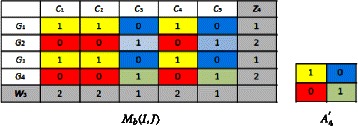

We compute $\left (EvalStab_{4}^{'},IndHomog_{4}^{'}\right)$, by using Eq. 4.3, we obtain:

$$\left(EvalStab_{4}^{'},IndHomog_{4}^{'}\right)=\left(0,\frac{0}{4}\right) $$

We compute (*z*_4_,*w*_4_,*A*_4_) starting from ($z_{4},w_{3},A_{4}^{'}$), by using Eq. 2.4, we obtain:

$$\left(z_{4},w_{4},A_{4}\right)=((1,2,1,2),(1,1,1,1,1),(1;0)) $$

We compute (*EvalStab*^4^,*IndHomog*^4^), by using Eq. 4.3, we obtain:

$$\left(EvalStab^{4},IndHomog^{4}\right)=(1,1) $$

Since we have

$${\kern30pt} \begin{aligned} (((z_{c},w_{c},A_{c})&\neq(z_{c-1},w_{c-1},A_{c-1}))\; \textbf{and} \;(EvalStab_{c},IndHomog_{c})\\ &\neq (EvalStab_{c-1},IndHomog_{c-1}))) \end{aligned} $$

**and**

$${\kern30pt} \begin{aligned} (((z_{c},w_{c},A_{c})&\neq(z_{c},w_{c-1},A_{c}^{'}))\,\,\textbf{and}\,\,((EvalStab_{c},IndHomog_{c})\\ &\neq (EvalStab_{(c-1)}^{'},IndHomog_{(c-1)}^{'})) \end{aligned} $$

we make another iteration

**Iteration 5:*c*=5**

We compute ($z_{5},w_{4},A_{5}^{'}$) starting from (*z*_4_,*w*_4_,*A*_4_), by using Eq. 2.4, we obtain:

$$\left(z_{5},w_{4},A_{5}^{'}\right)=((1,2,1,2),(1,1,1,1,1),(1;0)) $$

**Figure:**
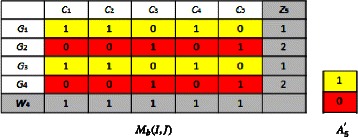

We compute $(EvalStab_{5}^{'},IndHomog_{5}^{'})$, by using Eq. 4.3, we obtain:

$$\left(EvalStab_{5}^{'},IndHomog_{5}^{'}\right)=(1,1) $$

We compute (*z*_5_,*w*_5_,*A*_5_) starting from ($z_{5},w_{4},A_{5}^{'}$), by using Eq. 2.4, we obtain:

$$\left(z_{5},w_{5},A_{5}\right)=((1,2,1,2),(1,1,2,1,2),(1,0;0,1)) $$

We compute (*EvalStab*^5^,*IndHomog*^5^), by using Eq. 4.3, we obtain:

$$\left(EvalStab^{5},IndHomog^{5}\right)=\left(0,\frac{0}{4}\right) $$

We have $(EvalStab_{4}^{'},IndHomog_{4}^{'}) = (EvalStab^{5},IndHomog^{5}))$ and (*z*_5_,*w*_5_,*A*_5_) $= (z_{4},w_{3},A_{4}^{'})$.

Since we have

$${\kern30pt} \begin{aligned} (((z_{c},w_{c},A_{c})&=(z_{c},w_{c-1},A_{c}^{'}))\,\,\textbf{and}\,\, ((EvalStab_{c},IndHomog_{c})\\ &= (EvalStab_{(c-1)}^{'},IndHomog_{(c-1)}^{'})) \end{aligned} $$

we stop the loop.

Then, we obtain (*z*_*opt*_,*w*_*opt*_,*A*_*opt*_)=(*z*_5_,*w*_5_,*A*_5_). Biclusters that contain only 0’s will not be considered because they represent genes that are not expressed under the related conditions. Finally, (*z*_*opt*_,*w*_*opt*_,*A*_*opt*_) can be represented in *M*_*b*_(*I,J*) as follows:

**Figure:**
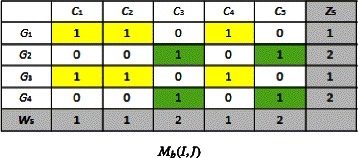

## Results for synthetic datasets

In this section, we present an experimental study to evaluate the performance of our algorithms of microarray data. Indeed, we compare the results of our algorithms to those obtained by a selection of known algorithms cited in the literature. We conducted experiments on synthetic and real datasets of microarrays. The idea behind testing on synthetic datasets is to investigate the ability of our algorithms to extract different types of biclusters. However, on real datasets, we seek to assess the degree of response of our algorithms for statistical and biological criteria.

### Synthetic microarray datasets and comparaison criteria

By adopting the strategy and data described in [1], we have experimented our algorithms on synthetic datasets by operarting as follows: First, we choose the number of biclusters, 3 clusters on rows (*g*=3) and 2 clusters on columns (*m*=2). Second, we use the *Latent Bernoulli Mixture* (LBM) model [1] to generate binary matrices (*mixtures*) by considering:

- (*a*) Overlapping biclusters (overlapping rate =5 *%* (well separated), 15 % (fairly separated) and 25 % (poorly separated)).
- (*b*) Different data sizes (matrix size =50×30 (small), 100×60 (medium) and 200×120 (large)).

Similar to [4], we use two indices, *Recovery* and *Relevance*, to evaluate our biclustering algorithms: Let *B*_1_ be a group of true implemented biclusters in a binary data matrix *M*_*b*_ and *B*_2_ be a group of output biclusters of a biclustering algorithm, *Relevance* reflects to what extent *B*_2_ is similar to *B*_1_, while *Recovery* quantifies how well each bicluster in *B*_1_ is recovered by *B*_2_ [3]:

(6.1) $$ Recovery=Overlap(B_{2},B_{1})  $$

(6.2) $$ Relevance=Overlap(B_{1},B_{2})  $$

where:

(6.3) $$ Overlap(B_{1},B_{2})=\frac{1}{|B1|}\underset{(I_{1},J_{1})\in B_{1}}{\sum}\underset{(I_{2},J_{2})\in B_{2}}{max}\frac{|I_{1}\cap I_{2}||J_{1}\cap J_{2}|}{|I_{1}\cup I_{2}||J_{1}\cup J_{2}|}  $$

We use also two other indices cited in [2],

(6.4) $$ Shared=\frac{S_{cb}}{Tot_{size}}100  $$

(6.5) $$ NotShared=\frac{S_{ncb}}{Tot_{size}}100  $$

where

*S*_*cb*_ is the volume of correctly extracted biclusters, *Tot*_*size*_ is the total volume of implemented biclusters and *S*_*NCB*_ is the volume of not correctly extracted biclusters.

The *Shared* index (resp. *NotShared*) represents the percentage of correctly (resp. not correctly) extracted biclusters with respect to all implemented biclusters in the data matrix. Indeed, when the *Shared* value is equal to 100 %, the algorithm extracts all the implemented biclusters. When the value of *NotShared* is 0 %, the algorithm extracts no cell outside the implemented biclusters.

### Experimental protocol

We have compared our algorithms to CC Cheng and Wee-Chung [2], OPSM Ben-Dor and Yakhini [5], ISA Ihmels et al. [4] and BiMax Kaiser and Leisch [6]. These algorithms were implemented in the *BIClustering Analysis Toolbox* (Bicat) platform. After several simulations, the parameters of our algorithms were set as listed in Table 1. Indeed, at each simulation, we set a parameter and we vary the other and vice inverse. Finally, we keep the parameters which give the nearest implemented biclusters in the starting template.

**Table 1:** Corresponding parameters values of our algorithms

Algorithms	Corresponding parameters values
*BiBinCons*	*minrow* = 2, *mincol* = 2
*BiBinAlter*	*minrow* = 2, *mincol* = 2

For CC, OPSM, ISA and BiMax algorithms, we keep the value of the default parameters values. Indeed, these values give biclusters of reasonable quality. We have adopted *Shared*, *NotShared*, *Recovery* and *Relevance* as comparaison criteria. Table 2 shows the best biclusters extracted by each algorithm:

**Table 2:** Values of *Shared* and *NotShared* for non overlapping biclusters

Algorithms	*Shared*	*NotShared*
CC	18.21 %	36.57 %
OPSM	46.39 %	74.42 %
ISA	39.38 %	5.31 %
BiMax	58.18 %	21.39 %
*BiBinCons*	88 %	12 %
*BiBinAlter*	100 %	37.03 %

As we can notice in Table 2, for the generated binary matrices, the best values of *Shared* and *NotShared* for non overlapping biclusters were obtained by the *BiBinAlter* algorithm. Indeed, to get a solution *B*_*opt*_, the combination between two biclusters provides additional volume for the conditions. This reasonnable additional volume is generated by a successive comparaisons between *P*_*max*_ and the other polynoms of *L*, and we locate the polynom *P*_*uncomon*_ that has the lowest number *ρ* of uncommon terms with *P*_*max*_. In fact, the interesting resulat is obtained because of we keep only the conditions that have not been removed by the pretreatment process. Besides, the extracted bicluster from the current matrix *M*_*b*_(*I,J*) is removed ans we set the cells of *M*_*b*_(*I,J*), representing the new bicluster, to 0. Table 3 shows the best biclusters extracted by each algorithm. As we can notice in Table 3, for the generated binary matrices, the best values of *Shared* and *NotShared* for overlapping biclusters were also obtained by the *BiBinAlter* algorithm. We can explain this as follows: *BiBinAlter* results covers most of the implemented biclusters. Table 4 presents the number of biclusters obtained bu our algorithms on real datastes.

**Table 3:** Values of *Shared* and *NotShared* for overlapping biclusters

Algorithms	*Shared*	*NotShared*
CC	13.21 %	36.57 %
OPSM	82.02 %	50.51 %
ISA	29.28 %	7.31 %
BiMax	48.18 %	22.39 %
*BiBinCons*	87.30 %	61 %
*BiBinAlter*	89.40 %	57.32 %

**Table 4:** Number of biclusters obtained by our algorithms on real datasets

Algorithms	*Yeast cell cycle*	*Human B-cell Lymphoma*
*EnumLat*	883	1921
*DecBinBicluster*	708	1720
*BiBinCons*	529	1900
*BiBinAlter*	881	1769
*RefineBicluster*	708	1700

## Results of our algorithms on real datasets

In this section, we evaluate our algorithms on real microarray datasets.

### Real microarray datasets

We have used two real microarray datasets: The Yeast cell cycle dataset which has been described and then pretreated in [1]. It contains the expression of 2884 genes in 17 terms ans the Human B-cell Lymphoma dataset which has been described by Alizadeh et al. [1], it contains 4026 genes and 96 conditions. These datasets are used frequently in the literature by biclustering algorithms.

### Experimental protocol

The first experiments concern the statistical validation. It enables to calculate the coverage for *Yeast cell cycle* and *Human B-cell Lymphoma* datasets and the *p*-value adjusted for*Human B-cell Lymphoma* datasets. The second experiments was applied to *Yeast cell cycle* in order to study the biological significance of extracted biclusters.

### Statistical validation

In order to validate statistically our algorithms on these real datasets, we evaluate the performance of *BiBinCons* and *BibinAlter*. We calculate the total number of cells covered by the biclusters. To do this, we have processed as in [2], and we have compared the results of our algorithms to those reported in [2]. In the literature, the coverage test was performed on *Yeast cell cycle* and *Human B-cell Lymphoma* datasets. This test is not applied to *RefineBicluster* algorithm because it is only a refinement algorithm.

Table 5 reports the percentage of *Coverage* on the different algorithms for *Yeast cell cycle* and *Human B-cell Lymphoma* datasets. We note that most algorithms have more or less close rates. For example, for the *Yeast cell cycle* datase, *BiBinCons* has the lowest performance. This is explained by the fact that *BiBinCons* extracts thousands of small sized biclusters. The CC algorithm extracts biclusters with random values. Thus, CC prohibits the genes/conditions already discovered to be selected in the next search process. This type of mask leads to a high coverage and preventing the discovery of large biclusters.

**Table 5:** Values of *Coverage* for *Yeast cell cycle* and *Human B-cell Lymphoma* datasets

Datasets	Algorithms	Total coverage	Genes coverage	Conditions coverage
Yeast celll cycle	CC	81.47 %	97.12 %	100 %
	*BiBinCons*	39.14 %	44.5 %	100 %
	*BiBinAlter*	47 %	48,03 %	100 %
Human B-cell Lymphoma	CC	36.81 %	91.58 %	100 %
	*BiBinCons*	34.14 %	37.51 %	100 %
	*BiBinAlter*	41 %	46.13 %	100 %

### Biological validation

To evaluate biologically extracted biclusters, we use the web tool *GOTermFinder*. To do this, we present the most significant shared biclusters. In this section, we evaluate *BiBinCons* and *BiBinAlter* algorithms on real microarray datasets. We have choosen this algorithm because it gaves the best results on synthetic datasets. Table 6 presents the most important terms of GO for the two most significant extracted biclusters from *Yeast cell cycle* dataset by *BiBinCons* and *BiBinAlter*.

**Table 6:** The most important terms of GO for the two most significant extracted biclusters from *Yeast cell cycle* dataset by *BiBinCons* and *BiBinAlter*

Biclusters	Biological process	Molecular function	Cell component
12 genes, 13 conditions	Cellular response to chromatin binding microtubule organizing 13 conditions DNA damage stimulus (25 %,0.00037) center part (66.7 %, 1:87 * 10-8) (16.7 %, 0.00742) response to DNA damage stimulus (66.7 %, 6:30 * 10-8) cellular response to stress (66.7 %, 2:12 * 10-7) cellular response to stimulus (66,7 %, 3:25 * 10-7) DNA repair (50 %, 2:58 * 10-5) response to stress (66.7 %, 2:98 * 10-5)	Chromatin binding microtubule organizing 13 conditions DNA damage stimulus (25 %,0.00037)	Microtubule organizing 13 conditions DNA damage stimulus (25 %,0.00037) center part (66.7 %, 1:87 * 10-8) (16.7 %, 0.00742)
11 genes, 11 conditions	Cell cycle process GTPase activator microtubule cytoskeleton 11 conditions (63.6 %, 2:93 * 10-5) activity (18.2 %,0.00994) (45.5 %, 6:33 * 10-6) cell cycle microtubule organizing (63.6 %, 6:85 * 10-5)	GTPase activator microtubule cytoskeleton 11 conditions (63.6 %, 2:93 * 10-5) activity (18.2 %,0.00994)	Microtubule cytoskeleton 11 conditions (63.6 %, 2:93 * 10-5) activity (18.2 %,0.00994) (45.5 %, 6:33 * 10-6) cell cycle microtubule organizing (63.6 %, 6:85 * 10-5) center (36.4 %,4:97 * 10-5) spindle pole body (36.4 %, 4:97 * 10-5) spindle pole (36.4 %, 6:77 * 10-5)

### Computing time

Table 7 shows the computing time of *BiBinCons* and *BiBinAlter* algorithms. All developed algorithms in this thesis were implemented in R under the R studio. The physical characteristics of the machine are as follows: a PC with an Intel Core 2 Duo T6400 with a clock frequency of 2.0 GHz and 3.5 GO of RAM. We note that *BiBinAlter* algorithm is the most time consuming and this is due to the use of proposed evaluation function.

**Table 7:** Computing time of our algorithms

Datasets	*BiBinCons*	*BiBinAlter*
*Yeast Cell Cycle*	32 min	37 min 12 sec
*Saccharomyces Cerevisiae*	8 min	8 min 3 sec

## Conclusion

In this paper, we have developed two biclustering algorithms of binary microarray data, called *BiBinCons* and *BiBinAlter*, adopting the *Iterative Row and Column Clustering Combination* (IRCCC) approach, however, the *BiBinAlter* algorithm is an improvement of *BiBinCons*. On the other hand, *BiBinAlter* differs from *BiBinCons* by the use of the *EvalStab* and *IndHomog* evaluation functions in addition to the *CroBin* one [1]. *BiBinAlter* can extract biclusters of good quality with better *p-values*. In this paper, we have presented an experimental study of our biclustering algorithms of microarray data. We have compared the results of our algorithms to those obtained by a selection of the known biclustering algorithms. We have conducted experiments on both synthetic and real datasets of microarrays. For both synthetic and real datasets, our biclustering algorithm *BiBinAlter* outperforms the other algorithms, followed by our other biclustering algorithms nd *BiBinCons*.
